# Correlation between overall survival and quality of life in colon cancer patients with chemotherapy

**DOI:** 10.1186/s12885-023-10989-x

**Published:** 2023-05-31

**Authors:** Yasuko Murakawa, Kazunori Ootsuka, Jun Kusaka, Kou Miura

**Affiliations:** 1 Department of Cancer Chemotherapy, Miyagi Cancer Center, Nodayama 47-1, Medeshima, Natori, Miyagi 981-1293 Japan; 2 Department of Gastroenterology, Miyagi Cancer Center, Natori, Japan; 3 Department of Digestive Surgery, Miyagi Cancer Center, Natori, Japan

**Keywords:** Chemotherapy, Hospitalisation, Inoperable colon cancer, Outpatient consultation, Overall survival, Quality of life

## Abstract

**Background:**

Patients presenting with inoperable colon cancer at first onset (ICF) or at time of relapse (ICR) are considered in unrecoverable. The therapeutic goal for unrecoverable cancer is to prolong overall survival (OS) and maintain a high quality of life (QOL). As data on objective indicators of QOL in cancer patients, such as length of hospitalisation (LOH), outpatient consultation times (OCT), and hospital-free survival (HFS), is limited, this study compared ICF and ICR with respect to OS and QOL over the entire clinical course.

**Methods:**

We retrospectively evaluated 90 inoperable colon cancer patients with chemotherapy and compared ICF and ICR in terms of OS, LOH, OCT, and HFS.

**Results:**

Patients with ICF had a worse OS than those with ICR. In patients with ICF and ICR, OS and LOH were not correlated but OS and OCT and OS and HFS were strongly correlated. In patients with ICF and ICR, OCT and HFS accounted for approximately 8% and 90% of their OS, respectively.

**Conclusions:**

The LOH, OCT, and HFS are important factors for evaluating objective QOL of patients with inoperable colon cancer and should be considered when making treatment decisions.

## Background

Inoperable colon cancer at first onset (ICF) or at time of relapse (ICR) is a fatal malignancy with poor prognosis. The 5-year survival is slightly greater than 10% in inoperable patients with stage IV colon cancer [1]. In recent years, new treatment strategies such as molecular-targeted therapies including angiogenesis and immune checkpoint inhibitors have been developed. However, curing inoperable colon cancers is challenging. Like other malignancies, colon cancer incidence increases with age. According to the latest mortality estimates for all cancer types, colon cancer ranks 2^nd^ in both the USA and Japan [2, 3].

In Japan, 30% of patients with colon cancer are diagnosed as inoperable stage IV at first onset, and 20% of patients experience relapse after curative operations [4]. The Adjuvant Colon Endpoint (ACCENT) data set showed that 35% of patients with colon cancer experience relapse after curative surgery, and the median time from relapse to death was 24 months [5]. In a meta-analysis, the median survival time (MST) of patients with inoperable and relapsed colon cancer was eight months without chemotherapy and 11.7 months with chemotherapy [6]. Several prognostic factors have been reported in colon cancer patients. However, these factors are insufficient for predicting each patient’s prognosis.

In inoperable colon cancer, the therapeutic goal is not achieving cure, but rather controlling symptoms, preventing complications, prolonging overall survival (OS), and maintaining a high quality of life (QOL). Many QOL questionnaires depend on patients’ subjective reports to evaluate QOL. However, it is quite a challenge to use these subjective questionnaires to evaluate QOL among patients in whom the condition progressively worsens.

Long-term hospitalisation and frequent outpatient consultations are undesirable to patients and can negatively impact QOL [7]. Length of hospitalisation (LOH), outpatient consultation times (OCT), and hospital-free survival (HFS), defined as the period without hospitalisation or outpatient consultation, could be useful objective indicators of QOL in cancer patients.

Our earlier studies report a strong correlation between OS and OCT/HFS in patients with inoperable oesophageal, pancreatic, and gastric cancers. And the choice of first-line chemotherapy affected the correlation between OS and OCT/HFS in patients with inoperable pancreatic cancers [8–10]. However, the data on comparison of colon cancer patients with ICF or ICR with respect to OS and QOL during the entire clinical course is lacking. Thus, this study compared OS, LOH, OCT, and HFS using ICF and ICR to clarify the relationship between OS and QOL.

## Methods

This study is a retrospective evaluation of 90 patients with inoperable colon cancer, visiting the Miyagi Cancer Center (Natori, Japan) between November 1, 2015, and November 30, 2020. All patients had a histological confirmation of adenocarcinoma from biopsy tissue collected by colonoscopy and Eastern Cooperative Oncology Group (ECOG) performance status (PS) [11] 0–2. Patients were diagnosed with inoperable colon cancer due to distant metastases or locally advanced tumours at the first onset or at the time of relapse after presumed curative surgery using computed tomography.

After diagnosis of inoperable colon cancer, all patients underwent chemotherapy. According to the National Comprehensive Cancer Network (NCCN) guidelines, the initial treatment recommended for patients was intensive therapy with FOLFOX/FOLFIRI ± bevacizumab/panitumumab or CAPEOX. Patients that were not recommended intensive therapy had 5-FU+leucovorin or Capecitabine. Patients positive for BRAF V600E mutation had FOLFOXIRI±bevacizumab [12].

We collected data on sex; age; ECOG PS; primary site and histology; RAS status; liver, lung, and peritoneal metastasis of locally advanced tumours, and on QOL factors OS, LOH, and OCT between November 1, 2015, and November 30,2020, from electronic medical records. OS was defined as the period from the beginning of the chemotherapy to the end of the observation, LOH as the total length of each hospitalisation, and OCT as the total number of outpatient visits.

The right-sidedness of primary site indicated cecal to transverse colon cancer and left-sidedness indicated descending to rectal colon cancer. Using computed tomography, we diagnosed peritoneal metastasis from ascites or peritoneal thickening, and a locally advanced tumours from direct infiltration into adjacent organs. Patients on palliative care who died at home were considered to have no LOH or OCT during their home stay. The exclusion criteria were patients undergoing 0–1 month of chemotherapy and missing data (OS, LOH, and OCT).

## Statistical analyses

We used multiple logistic regression analysis to compare ICF to ICR with respect to clinicopathological characteristics (e.g., sex; age; ECOG PS, primary site; histology; RAS status; liver, lung, and peritoneal metastasis; and locally advanced tumour). The OS curves were estimated using the Kaplan–Meier method and compared using the log-rank test. Multivariate Cox regression analysis was performed to adjust for confounding factors of OS. A two-tailed *p*-value of < 0.05 was considered statistically significant.

The correlation between OS and LOH/OCT/HFS was examined using scatter plot analysis, and ICF and ICR were compared. A coefficient of determination (COD), r2≥0.5, was considered a strong correlation, while 0.5>r2≥0.1 was considered a moderate correlation. All statistical analyses were performed using Statistical Package for the Social Sciences for Windows (software version 27, SPSS Inc., Chicago, IL, USA).

## Results

Ninety patients with unrecoverable colon cancer were enrolled in the study (Table 1). There were more men than women (male:female ratio, 54:36), and one-fourth of the patients were older (>70, ≤ 70 years; 24:66). Patients with an ECOG PS of 1 accounted for 50% of the total cohort (PS0, PS1, PS2; 28, 50, 12). Almost 80% of patients had left-sided colon cancer (right vs. left, 16:74). Almost 60% of patients had moderately differentiated adenocarcinoma (well- vs. moderately- vs. poorly differentiated, 25:58:7) Almost half of patients had RAS mutant (RAS wild vs. mutant, 43:47). Almost 70% of patients had liver metastases (±, 59/31), one-third of patients had lung metastases (±, 30/60) and few patients had peritoneal metastases and a locally advanced tumour (peritoneal metastasis ±, 9/81; locally advanced tumour ±, 13/77). Significant and independent characteristic difference was not observed between ICF and ICR.

**Table 1:** Multivariate logistic regression analysis of patients with inoperable or relapsed colon cancer

			ICF		ICR		OR(95%CI)		*P-*value
Variable	n	(%)	n	(%)	n	(%)			
sex									
female	36	(40.0)	32	(45.1)	4	(21.1)	0.44	(0.11–1.72)	0.238
male	54	(60.0)	39	(54.9)	15	(78.9)		1.00(ref.)	
age (years)									
≦70	66	(73.3)	52	(73.2)	14	(73.7)	1.21	(0.28–5.33)	0.799
>70	24	(26.7)	19	(26.8)	5	(26.3)		1.00(ref.)	
ECOG PS									
0	28	(31.1)	17	(23.9)	11	(57.9)	9.94	(0.88–111.88)	0.063
1	50	(55.6)	43	(60.6)	7	(36.8)	1.81	(0.18–18.42)	0.617
2	12	(13.3)	11	(15.5)	1	(5.3)		1.00(ref.)	
primary site									
right-sided	16	(17.8)	13	(18.3)	3	(15.8)	0.91	(0.13–6.50)	0.925
left-sided	74	(82.2)	58	(81.7)	16	(84.2)		1.00(ref.)	
histology									
well differentiated	25	(27.8)	20	(28.2)	5	(26.3)	1.58	(0.08–29.91)	0.761
moderately differentiated	58	(64.4)	45	(63.4)	13	(68.4)	1.91	(0.13–27.47)	0.635
poorly or undifferentiated	7	(7.8)	6	(8.5)	1	(5.3)		1.00(ref.)	
RAS									
wild	43	(47.3)	30	(42.3)	13	(68.4)	3.16	(0.83–11.95)	0.092
mutant	47	(51.6)	41	(57.7)	6	(31.6)		1.00(ref.)	
metastasis									
liver									
(-)	31	(34.4)	21	(29.6)	10	(52.6)	4.47	(0.98–20.42)	0.053
(+)	59	(65.6)	50	(70.4)	9	(47.4)		1.00(ref.)	
lung									
(-)	60	(66.7)	47	(66.2)	13	(68.4)	2.27	(0.46–11.23)	0.313
(+)	30	(33.3)	24	(33.8)	6	(31.6)		1.00(ref.)	
peritoneum									
(-)	81	(90.0)	63	(88.7)	18	(94.7)	8.55	(0.52–139.56)	0.132
(+)	9	(10.0)	8	(11.3)	1	(5.3)		1.00(ref.)	
locally advanced tumour									
(-)	77	(85.6)	60	(84.5)	17	(89.5)	3.99	(0.44–35.83)	0.217
(+)	13	(14.4)	11	(15.5)	2	(10.5)		1.00(ref.)	
total	90	(100)	71	(100)	19	(100)			

*CI* Confidence interval, *ECOG* Eastern Cooperative Oncology Group, *ICF* Inoperable colon cancer at first onset, *ICR* Inoperable colon cancer at time of relapse, *OR* Odds ratio, *PS* Performance status, *ref* Reference

**Fig. 1:**
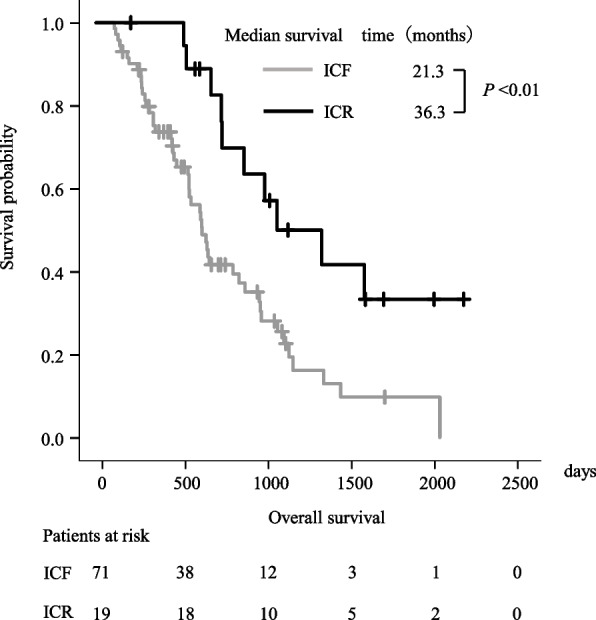
Kaplan–Meier curves for overall survival

As shown in Table 2, in the analysis of each variable, MST showed significant difference between ICF and ICR (21.3 vs. 36.3 months, *p*<0.01)) (Fig. 1), by sex (female: 18.7 vs. male: 23.1 months,* p*<0.05), and RAS wild and mutation (25.9 vs. 18.9 months,* p*<0.05). However, locally advanced tumours were significantly and independently associated with poor OS compared to non-locally advanced tumours (hazard ratio [HR]:0.27, 95% [Confidence Interval (CI)]:0.11–0.65; *p*<0.005) (Table 3).

**Table 2:** Median survival time from Kaplan–Meier curves

	MST (months)	SD (months)	*P-*value
variable			
timing diagnosed as inoperable			<0.01^*^
ICF	21.3	1.8	
ICR	36.3	9.4	
sex			<0.05^*^
female	18.7	1.6	
male	23.1	2.6	
age (years)			0.691
≦70	21.3	2.8	
>70	22.5	4.6	
ECOG PS			0.066
0	29.9	4.9	
1	18.7	3.3	
2	20.8	12.1	
primary site			0.676
right-sided	28.8	12.3	
left-sided	21.3	1.9	
histology			0.651
well differentiated	23.1	6.4	
moderately differentiated	22.1	2.3	
poorly or undifferentiated	18.5	1.0	
RAS			<0.05^*^
wild	25.9	6.0	
mutant	18.9	1.9	
metastasis			
liver			0.153
(-)	21.2	7.5	
(+)	22.1	2.4	
lung			0.592
(-)	22.8	2.8	
(+)	16.2	2.5	
peritoneum			0.383
(-)	22.1	1.9	
(+)	18.6	4.5	
locally advanced tumour			0.415
(-)	22.5	2.0	
(+)	18.5	6.6	

*ECOG* Eastern Cooperative Oncology Group, *ICF* Inoperable colon cancer at first onset, *ICR* Inoperable colon cancer at time of relapse, *MST* Median survival time, *PS* performance status

^*^<0.05

**Table 3:** Overall survival using Cox regression analysis

	Overall survival	
	HR (95.0% CI)	*P*-value
ICF vs. ICR	2.19 (1.00–4.82)	0.052
SEX(female vs. male)	1.33(0.74–2.38)	0.347
age (≦70 vs.>70 years)	0.98(0.50–1.92)	0.954
site(right vs. left)	0.42(0.17–1.02)	0.054
histology		
well differentiated vs. poorly or undifferentiated	0.47(0.15–1.51)	0.205
moderately differentiated. vs. poorly or undifferentiated	0.45(0.16–1.31)	0.142
RAS (wild vs. mutant)	0.55(0.29–1.03)	0.062
ECOG PS		
0 vs. 2	1.85(0.68–5.01)	0.225
1 vs. 2	1.92(0.79–4.67)	0.149
liver metastasis (-) vs. (+)	0.66(0.32–1.40)	0.280
lung metastasis (-) vs. (+)	0.84(0.42–1.67)	0.610
peritoneal metastasis (-) vs. (+)	0.47(0.17–1.32)	0.152
Locally advanced tumour (-) vs. (+)	0.27(0.11–0.65)	<0.005^*^

*CI* Confidence interval, *ECOG* Eastern Cooperative Oncology Group, *HR* Hazard ratio, *ICF* Inoperable colon cancer at first onset, *ICR* Inoperable colon cancer at time of relapse, *PS* Performance status

^*^<0.05

**Fig. 2:**
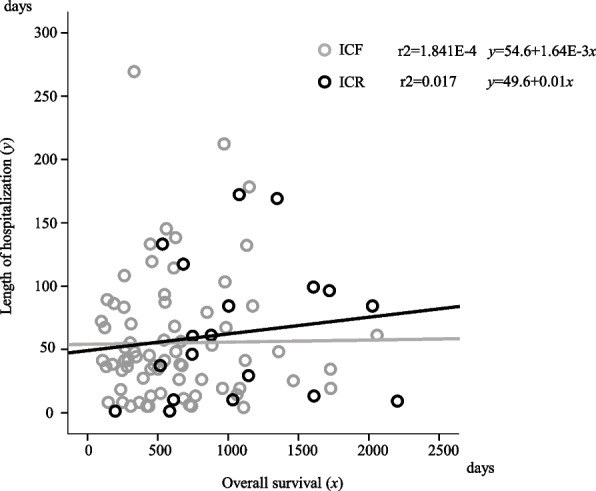
Correlation between overall survival and length of hospitalisation

Significant correlation was not observed for OS and LOH in ICF and ICR (COD: r2=1.841E-4 and 0.017 respectively) (Fig. 2).

**Fig. 3:**
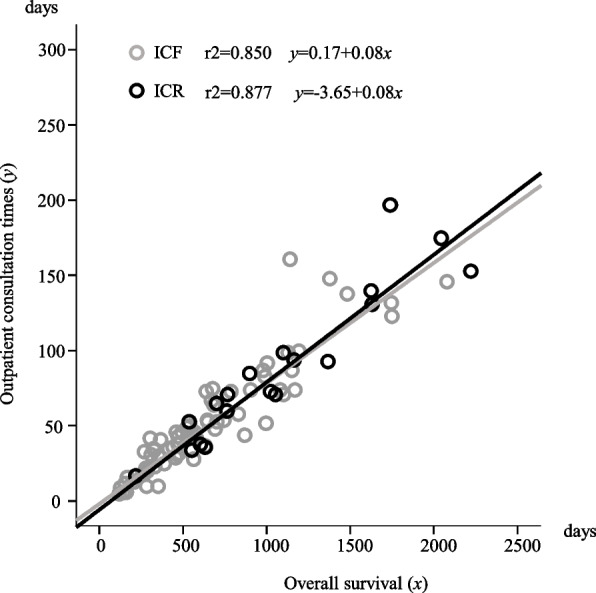
Correlation between overall survival and outpatient consultation times

**Fig. 4:**
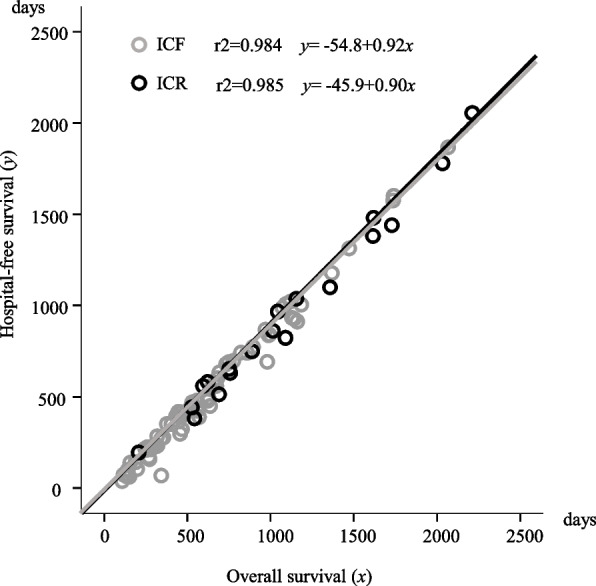
Correlation between overall survival and hospital-free survival

In determining correlation between OS and OCT, strong correlation between OS (x-axis) and OCT (y-axis) was observed between ICF and ICR (COD: r2=0.850, y=0.17+0.08x and r2=0.877, y=-3.65+0.08x, respectively). This result indicates that OCT accounted for 8% of the patients’ OS period (Fig. 3). In determining correlation between OS and HFS, strong correlation was observed between OS (x-axis) and HFS (y-axis) among patients with ICF and ICR (COD: r2=0.984, y=-54.8+0.92x and r2=0.985, y=-45.9+0.90x, respectively). This indicated that HFS made up almost 90% of patients’ OS (Fig. 4).

Though detailed data were not shown; 6.7% (6/90) of patients died at home while receiving palliative home care, and the average number of days at home was 32.7 days (SD 24.0).

## Discussion

This study showed that patients with ICF had worse OS than those with ICR. Patients with unrecoverable colon cancer attributed approximately 8% and 90% of their OS period to OCT and HFS, respectively, regardless of ICF and ICR.

Many prognostic factors are reported in colon cancer, such as primary sites [13], histology [14], metastatic sites [15], and RAS mutations [16]. The T stage that indicates the histological depth of cancer invasion is reported to best indicate the prognosis of OS [17]. Tumour with advanced T stage: T4b indicates the direct invasion into other organs. Our results showed that locally advanced tumours showing direct invasion into other organs were significantly and independently associated with poor prognosis. The ICF group had slightly more locally advanced tumours than the ICR group (ICF, 15.5%; ICR, 10.5%).

Using the Surveillance Epidemiology and End Results (SEER) database, MST of stage IV colon cancer was reported to be 13 months [1]. One study showed that the MST after relapse of patients with resected stage III colon cancer varied from nine to 35 months depending on genetic specificities [18]. Our MST results (ICF, 21.3 months; ICR, 36.3 months) were almost comparable with those of previous studies.

The World Health Organization (WHO) proposed the WHOQOL-BREF, that contains a total of 26 questions instead of the WHOQOL-100, which may be too lengthy for practical use. These questionnaires contain four domains: physical health, psychological state, social relationships, and the environment [19]. Commonly used QOL questionnaires for cancer patients are the European Organization for Research and Treatment of Cancer QLQ-C30 with 30 questions and the Functional Assessment of Cancer Therapy-General with 27 questions [20, 21]. The feasibility of a longitudinal QOL survey using a questionnaire has been reported [22]. However, QOL evaluation was often difficult to analyse because of missing data in the questionnaire [23].

In this study, the main reasons for hospitaliszation were as follows: (1) implantation or defect of the central venous port system [24]; (2) first-line chemotherapy, and (3) to help control symptoms that are difficult to manage with OCT. Outpatient consultations were conducted for (1) subsequent chemotherapy cycles, (2) evaluation of treatment outcomes using techniques, such as endoscopy and computed tomography, and (3) identifying ways to manage the exacerbation of cancer and adverse effects of chemotherapy. Long hospitalisation and frequent outpatient consultation adversely affect QOL.

Older patients with higher comorbidities are more likely to be hospitalised while undergoing chemotherapy [25]. Hospitalisation exacerbates disruptions to circadian rhythms and impairs QOL [26]. Waiting time during outpatient consultation is generally identified as a factor that affects patient satisfaction, thus affecting QOL [27]. Unplanned outpatient consultations due to adverse effects of chemotherapy were reported to affect QOL and subsequent treatment [28, 29]. Patients with colon cancer reportedly have worse physical and mental QOL during chemotherapy than the general population [30]. Therefore, cancer patients should demand information related to adverse effects of chemotherapy [31]. Physicians prefer treatments that maximise survival times, even if these treatments have severe adverse effects [32].

This study had several limitations. We conducted a retrospective study of 90 patients from a single facility; thus, the number of ICR was small. We excluded data on home palliative care. The HFS is insufficient in evaluating QOL during the entire clinical course because it is not a QOL indicator based on the patient’s own evaluation.

## Conclusions

Patients with ICF may have worse OS than those with ICR. Patients with unrecoverable colon cancer showed a strong correlation between OS and OCT/HFS, regardless of ICF and ICR, which was similar to other unrecoverable cancers, such as oesophageal, gastric, and pancreatic cancers. When conducting a randomised controlled trial and clinical practice study, it may be necessary to examine OCT and HFS to evaluate the QOL for the entire clinical course.

## Data Availability

Raw data for this study are shown in Fig share (10.6084/m9.figshare.19769041.v1).
